# Supplementary material for: Orally delivered toxin–binding protein protects against diarrhoea in a murine cholera model
Source: Nat Commun. 2025 Mar 19;16:2722. doi: 10.1038/s41467-025-57945-w (PMC11923127; doi:10.1038/s41467-025-57945-w)
Supplement: Supplementary file 1 — Supplementary Information [file 41467_2025_57945_MOESM1_ESM.pdf]

Orally delivered toxin–binding protein protects against diarrhoea in a murine cholera model

Marcus Petersson[1,2*], Franz G. Zingl[3,4*], Everardo Rodriguez-Rodriguez[2], Jakob K.H. Rendsvig[2], Heidi Heinsøe[2], Emma Wenzel Arendrup[2], Natalia Mojica[5], Dario Segura Peña[6], Nikolina Sekulić[6,7], Ute Krengel[5], Monica L. Fernández-Quintero[1], Timothy P. Jenkins[1], Lone Gram[1], Matthew K. Waldor[3,4], Andreas H. Laustsen[1,2†], Sandra Wingaard Thrane[2†]

[1] Department of Biotechnology and Biomedicine, Technical University of Denmark, Kongens Lyngby, Denmark

[2] Bactolife A/S, Copenhagen, Denmark

[3] Department of Immunology and Infectious Diseases, Harvard T. H. Chan School of Public Health, Boston, MA, USA

[4] Division of Infectious Diseases, Brigham and Women's Hospital, Boston, MA, USA

[5] Department of Chemistry, University of Oslo, Oslo, Norway

[6] Centre for Molecular Medicine Norway, University of Oslo, Oslo, Norway

[7] Department of Molecular Medicine, Institute of Basic Medical Sciences, Faculty of Medicine, University of Oslo, Oslo, Norway

*These authors contributed equally: Marcus Petersson, Franz G. Zingl

†Correspondence and requests for materials should be addressed to A.H.L. (email: ahola@bio.dtu.dk) or to S.W.T. (email: swt@bactolife.com)

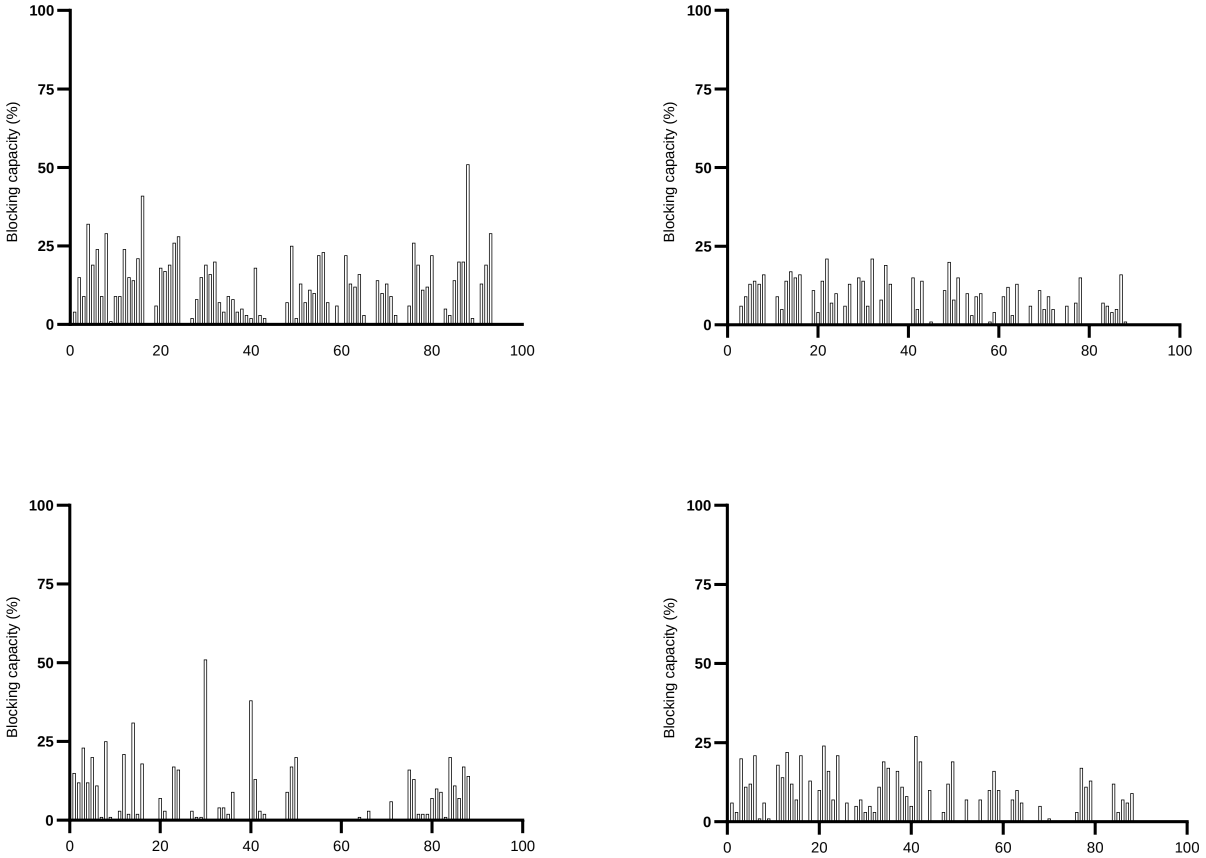

Supplementary Figure 1. The ability of 380 CTXB-specific monovalent V_HHs expressed by *E. coli* to block the CTXB–GM1 interaction. V_HHs in overnight culture supernatants of individual *E. coli* clones were incubated with biotinylated CTXB and added to GM1 immobilized on a microtiter plate. The blocking capacity of the V_HHs was normalized against a CTXB-only control. Each *E. coli* supernatant containing V_HHs was analysed once. Source data are provided as a Source Data file.

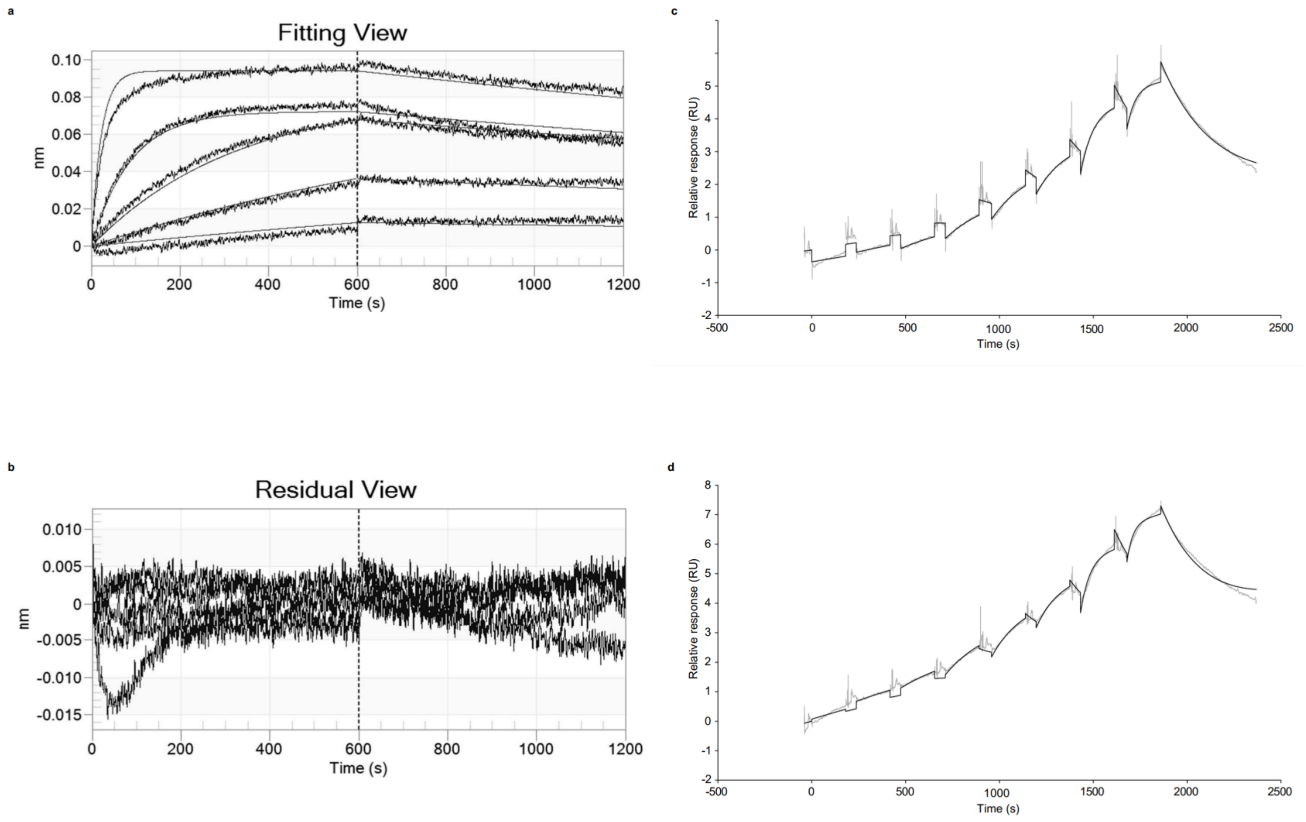

Supplementary Figure 2. CTXB affinity determination for BL3.1 using bio-layer interferometry (BLI) and surface plasmon resonance (SPR). a BLI sensorgrams obtained by using BL3.1 as the ligand (76 nM) and CTXB the analyte in serial dilutions (120–0.470 nM). The signal from the non-CTXB control was subtracted from each sample and the data fitted to a global model (1:1 binding sites). **b** Residual plots showing the deviation of the experimental data from the theoretical fitting. **c**,**d** SPR sensorgrams for the kinetic interaction between immobilized CTXB (5 µg ml^{-1}) and the analyte BL3.1 (1–500 nM), analysed in duplicates. Data fitted to a global model (1:1 binding sites).

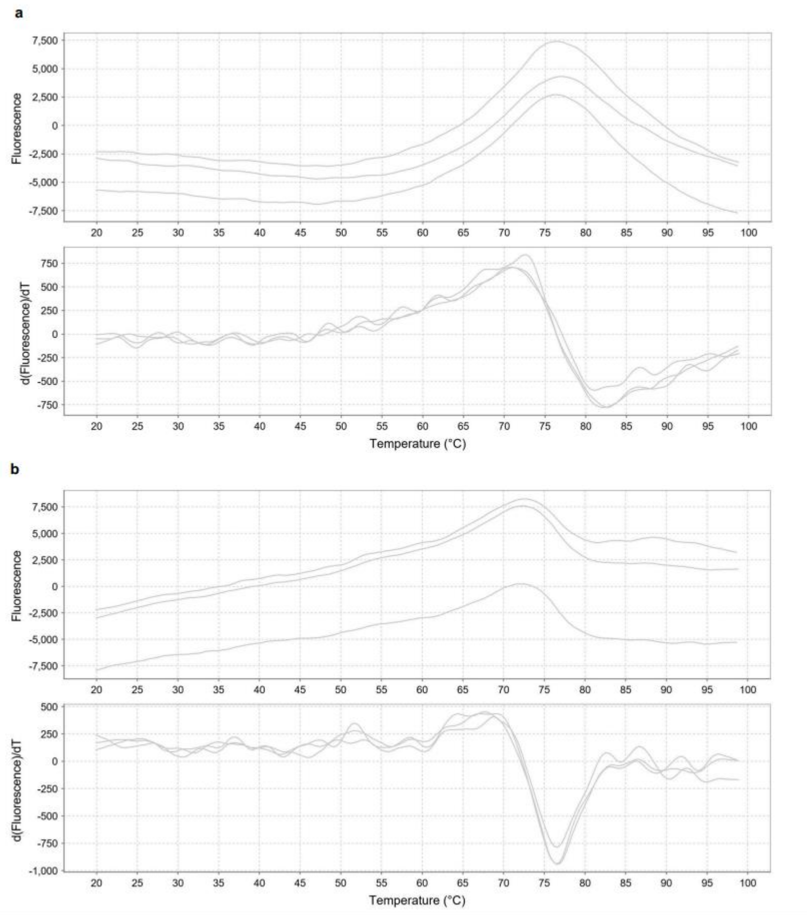

Supplementary Figure 3. Melt curve plots for BL3.2 and CTX. a Melt curve plots of the fluorescence and derivative fluorescence data obtained by analysing the thermal stability of BL3.2 using differential scanning fluorimetry (Protein Thermal Shift™). Analysis performed in triplicates. **b** Melt curve plots of the fluorescence and derivative fluorescence data obtained by analysing the thermal stability of CTX using differential scanning fluorimetry (Protein Thermal Shift™). Analysis performed in triplicates.

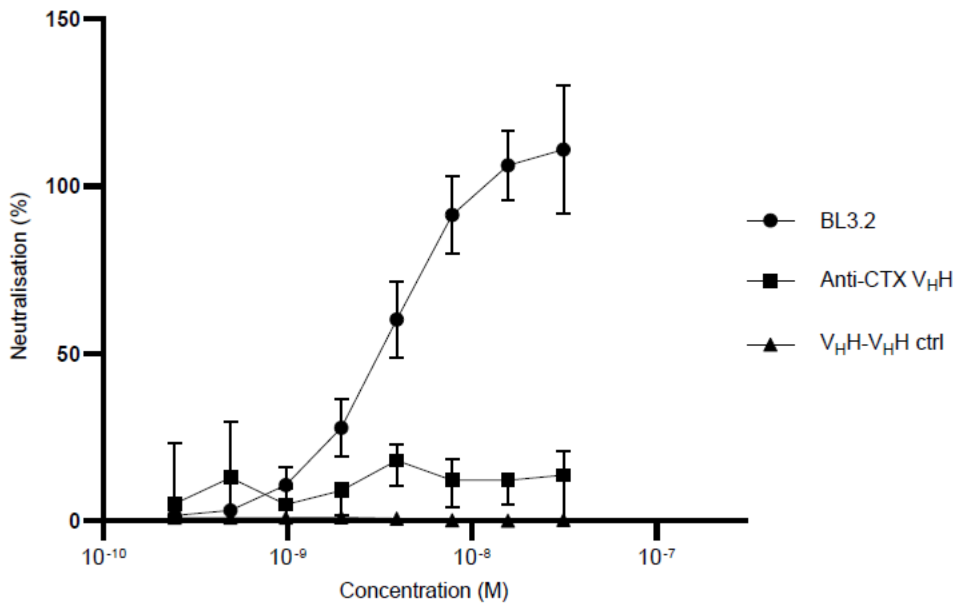

Supplementary Figure 4. Neutralization of CTX interaction with the GM1 receptor on human colon adenocarcinoma (HCA-7) cells. Neutralization of CTX (0.115 nM) by increasing concentrations (0.240–31.25 nM) of the bivalent V_HH construct BL3.2, a previously reported monomeric anti-CTX V_HH, or a non-specific bivalent V_HH construct control[43]. For BL3.2 and the non-specific control, each point represents interpolated mean values from biological duplicates comprised of three technical replicates. The anti-CTX V_HH was analysed once (in technical triplicates). Error bars represent standard deviation. Source data are provided as a Source Data file.

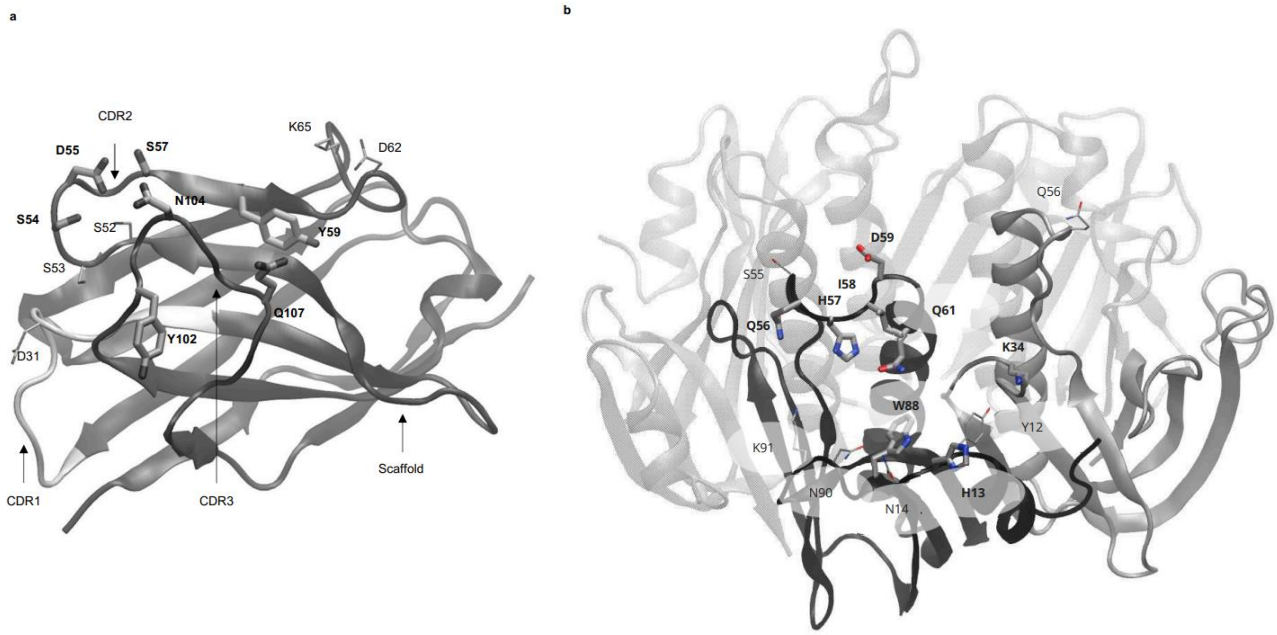

Supplementary Figure 5. In silico analysis of the BL3.2 paratope binding to CTXB using the EpiC platform (v. 9, Raven Biosciences). a The four primary residues in the BL3.2 complementarity-determining region two (CDR2) (Ser54, Asp55, Ser57, and Tyr59) and three primary residues in the CDR3 (Tyr102, Asn104, and Gln107) predicted to be crucial for interaction with CTX are highlighted in bold. Four supporting residues were found in the CDR1 (Asp31), the CDR2 (Ser52), and the antibody scaffold (Asp62 and Lys65). **b** The CTXB epitope (black) interacting with BL3.2, with the adjacent monomer (dark grey) and the three remaining CTXBs (light grey) depicted. Eight primary residues (in bold) were identified, seven (His13, Gln56, His57, Ile58, Asp59, Gln61, and Trp88) in the same monomeric subunit and one (Lys34) in the adjacent monomer. In addition, five supporting residues (Tyr12, Asn14, Ser55, Asn90, and Lys91) were identified.

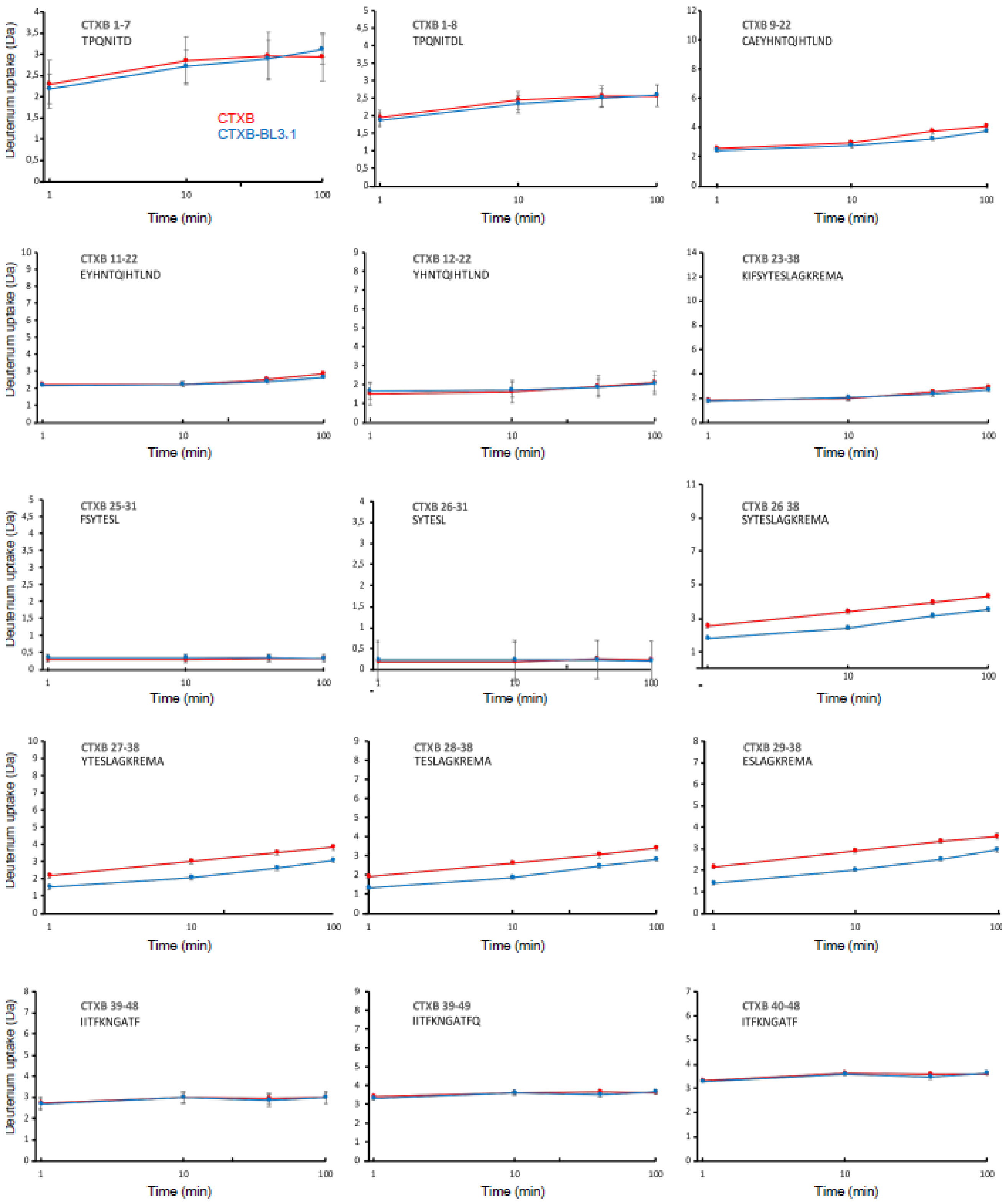

Supplementary Figure 6. Hydrogen–deuterium exchange mass spectrometry (HDX-MS) epitope mapping of amino acid positions 1–49 of CTXB. Deuterium uptake plots for amino acids in positions

1–49 of CTXB (red) or the CTXB–BL3.1 complex (blue). Each time point (1, 10, 40 and 100 min) was analysed in technical triplicates. Data are presented as mean values with standard deviation.

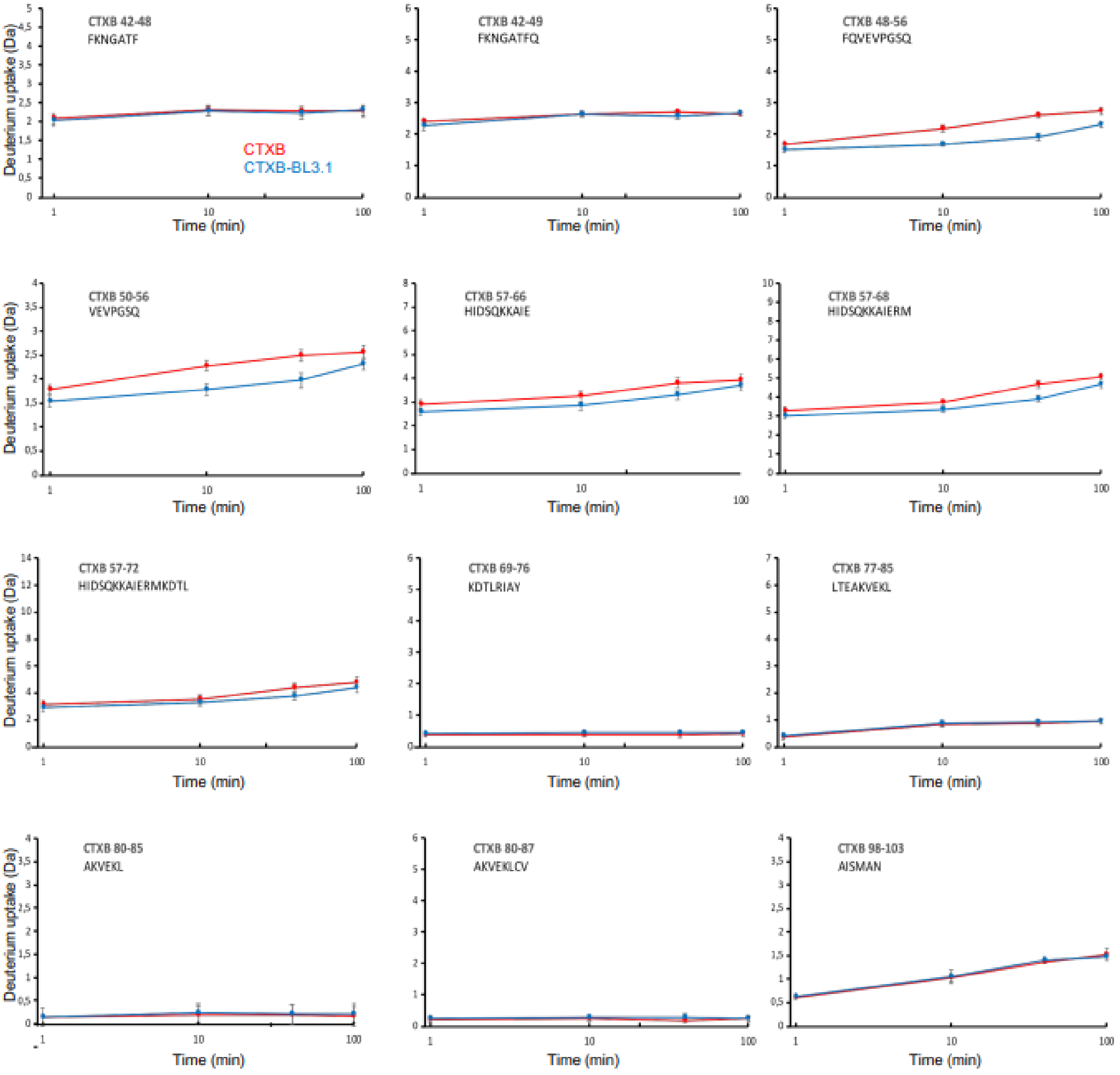

Supplementary Figure 7. Hydrogen–deuterium exchange mass spectrometry (HDX-MS) epitope mapping of amino acid positions 42–103 of CTXB. Deuterium uptake plots for amino acids in positions 42–103 of CTXB (red) or the CTXB–BL3.1 complex (blue). Each time point (1, 10, 40 and

100 min) was analysed in technical triplicates. Data are presented as mean values with standard deviation.

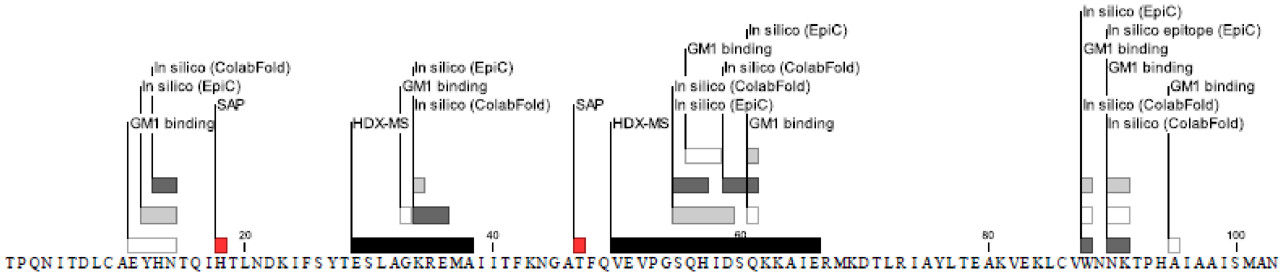

Supplementary Figure 8. Structural analysis of BL3.2–CTX interaction in relation to GM1 receptor–binding pocket. Comparison of machine learning simulations (ColabFold and EpiC) of the CTXB epitope predicted to participate in BL3.2 binding against hydrogen–deuterium exchange mass spectrometry (HDX-MS) analysis. The CTXB genotype displayed (*ctxB7*) has been annotated with the previously reported GM1–binding site and two (positions 18 and 47) single-amino acid polymorphism (SAP) sites to represent the three toxin genotypes (*ctxB1*, *ctxB3*, and *ctxB7*) that have caused the current and all previous cholera pandemics[11,34,35].

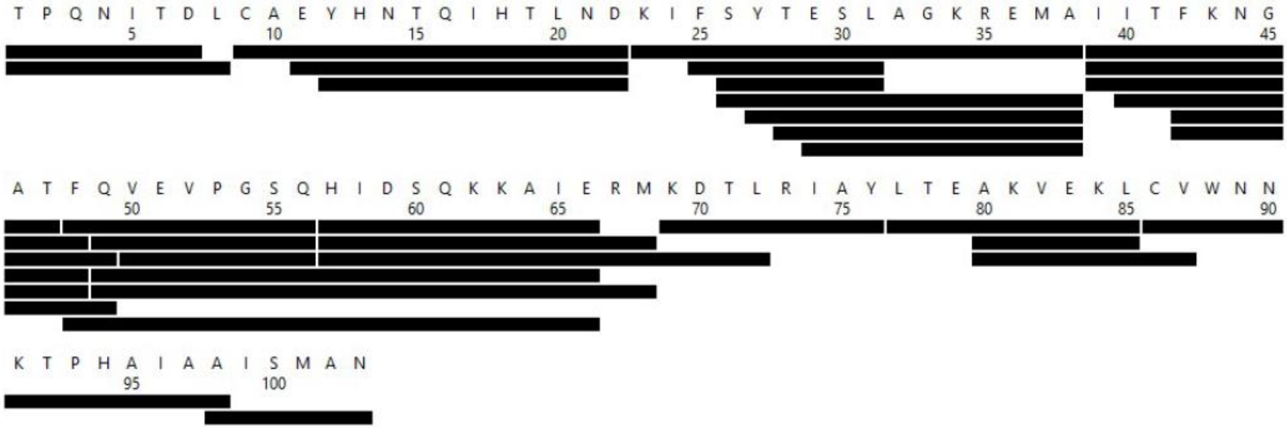

Supplementary Figure 9. CTX coverage prior to hydrogen–deuterium exchange mass spectrometry (HDX-MS) analysis of the BL3.1–CTX interaction. HDX-MS coverage map for

CTXB. Each rectangle represents a unique peptide. The obtained peptide from the non-deuterated samples covers 100 % of CTXB with an average redundancy of three peptides per amino acid.

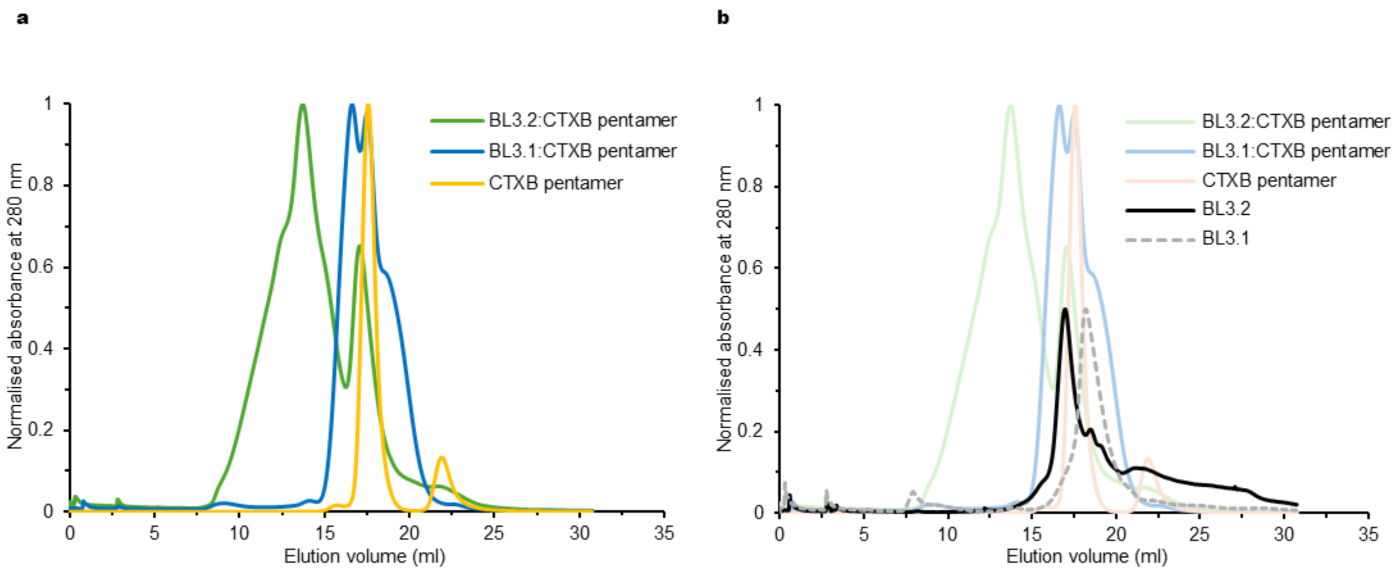

Supplementary Figure 10. Complex formation studies by size-exclusion chromatography (SEC). **a** Superimposition of SEC profiles of CTXB (yellow), in complex with either BL3.1 (blue) or BL3.2 (green). BL3.1 and BL3.2 were analysed at a 2:5 and 1:5 binding site ratio (V_HH construct:CTXB pentamer), respectively. Absorbance at 280 nm was normalised to the maximum peak absorbance in each chromatogram. **b** SEC profiles of BL3.2 (black bold line) and BL3.1 (grey dashed line) superimposed on chromatograms from panel **a**. To aid in visual presentation, absorbance of the BL3.2 and BL3.1 chromatograms was halved.

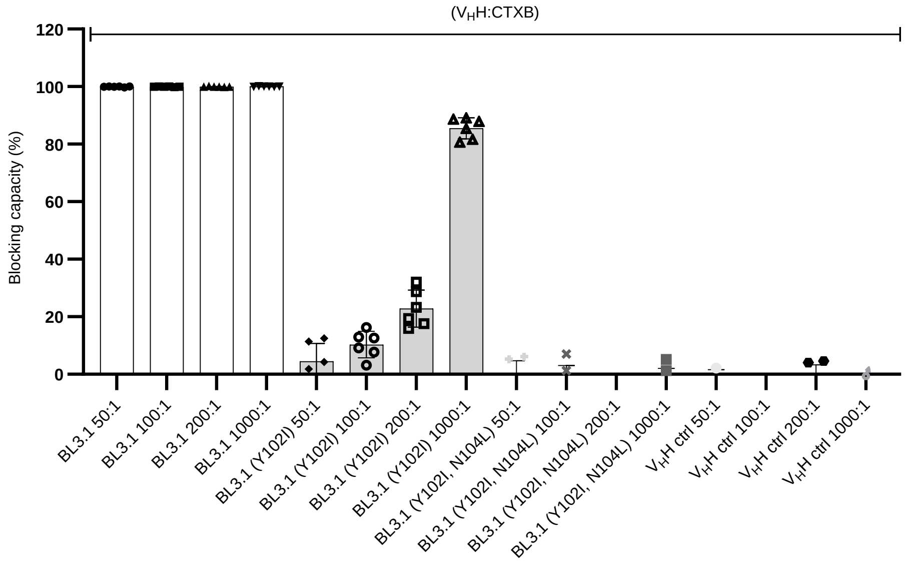

Supplementary Figure 11. Experimental verification of in silico predictions of key amino acids in the BL3.2 complementarity-determining region three (CDR3) paratope. The impact of amino acid substitutions (Y102I and N104L) in the predicted BL3.1/BL3.2 paratope on CTXB–GM1-blocking capacity. The average blocking capacity of BL3.1 or two variants thereof was determined at molar ratios between 50:1 and 1000:1 (V_HH:CTXB), based on duplicate measurements of technical triplicates. Error bars represent standard deviation. A monomeric V_HH construct without specificity for CTXB was included as a control. Source data are provided as a Source Data file.

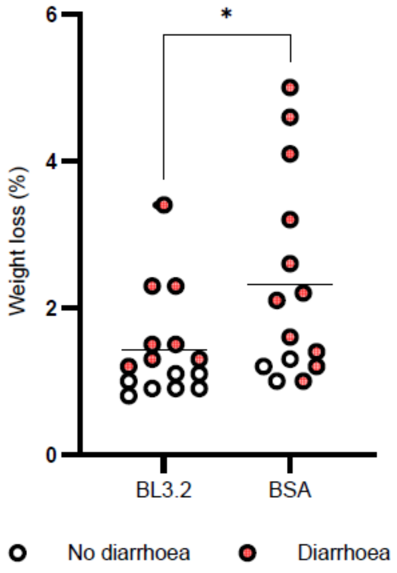

Supplementary Figure 12. Weight loss of infant mice orally administered a mixture of BL3.2 and CTX. Weight loss for 5-day-old CDI infant mice (Charles River Laboratories, strain 022, mixed sex) 16 h after oral administration (50 µl) of BL3.2 (9 mg ml^{-1}) pre-incubated at 37 °C for 30 min with CTX (10 mg ml^{-1}), compared to a bovine serum albumin (BSA) (9 mg ml^{-1}) control group. Statistical difference (*$P < 0.05$) in weight loss between the BL3.2 group (n = 15) and the BSA group (n = 14) calculated using the two-tailed Mann–Whitney U test ($P = 0.0395$), with the average indicated by the horizontal line. Diarrhoeal onset (red) or diarrhoeal absence (white) was visually monitored up until the experiment was terminated. Source data are provided as a Source Data file.

		**BLI**			**SPR**		
**Ligand**	**Analyte**	**k_{on} ($M^{-1} s^{-1}$)**	**k_{off} (s^{-1})**	**K_D (nM)**	**k_{on} ($M^{-1} s^{-1}$)**	**k_{off} (s^{-1})**	**K_D (nM)**
BL3.1	CTXB	$3.69 \times 10^{5} \pm 0.03$	$2.80 \times 10^{-4} \pm 0.04$	0.76 ± 0.13	N/A	N/A	N/A
CTXB	BL3.1	N/A	N/A	N/A	$4.59 \times 10^{4} \pm 0.78$	$3.86 \times 10^{-3} \pm 0.33$	85.50 ± 7.30

Supplementary Table 1. Characterization of BL3.1 affinity towards CTXB using bio-layer interferometry (BLI) and surface plasmon resonance (SPR).

Model	pLDDT	PAE	Number of clusters	Electrostatic interaction energies (kcal mol^{-1})	VdW interaction energies (kcal mol^{-1})
1	77.4	14.6	86	-104 ± 77	-38 ± 22
2	77.5	15.0	7	-178 ± 68	-71 ± 15
3	81.8	14.1	3	-347 ± 117	-68 ± 10
4	77.6	15.0	3	-195 ± 42	-85 ± 7

Supplementary Table 2. The four potential binding poses (Model 1–4) between BL3.2 and CTXB identified via ColabFold, including the AlphaFold2 confidence measures. The predicted local difference test (pLDDT) and predicted aligned error (PAE) are averages based on two repetitions of 1 µs each. The number of clusters were identified with a root mean square deviation (RMSD) cut-off of 5 Å. Interaction energies, electrostatic and van deer Waals, are displayed as the average of two repetitions with standard deviation.
