# Supplementary material for: Orally delivered toxin–binding protein protects against diarrhoea in a murine cholera model
Source: Nat Commun. 2025 Mar 19;16:2722. doi: 10.1038/s41467-025-57945-w (PMC11923127; doi:10.1038/s41467-025-57945-w)
Supplement: Supplementary file 2 — Reporting Summary [file 41467_2025_57945_MOESM2_ESM.pdf]

nature portfolio

Corresponding author(s): SWT and AHL

Last updated by author(s): Jan 15, 2025

Reporting Summary

Nature Portfolio wishes to improve the reproducibility of the work that we publish. This form provides structure for consistency and transparency in reporting. For further information on Nature Portfolio policies, see our Editorial Policies and the Editorial Policy Checklist.

Statistics

For all statistical analyses, confirm that the following items are present in the figure legend, table legend, main text, or Methods section.

n/a	Confirmed	
☐	☒	The exact sample size (*n*) for each experimental group/condition, given as a discrete number and unit of measurement
☐	☒	A statement on whether measurements were taken from distinct samples or whether the same sample was measured repeatedly
☐	☒	The statistical test(s) used AND whether they are one- or two-sided<br>*Only common tests should be described solely by name; describe more complex techniques in the Methods section.*
☒	☐	A description of all covariates tested
☒	☐	A description of any assumptions or corrections, such as tests of normality and adjustment for multiple comparisons
☐	☒	A full description of the statistical parameters including central tendency (e.g. means) or other basic estimates (e.g. regression coefficient) AND variation (e.g. standard deviation) or associated estimates of uncertainty (e.g. confidence intervals)
☐	☒	For null hypothesis testing, the test statistic (e.g. *F*, *t*, *r*) with confidence intervals, effect sizes, degrees of freedom and *P* value noted<br>*Give P values as exact values whenever suitable.*
☒	☐	For Bayesian analysis, information on the choice of priors and Markov chain Monte Carlo settings
☒	☐	For hierarchical and complex designs, identification of the appropriate level for tests and full reporting of outcomes
☒	☐	Estimates of effect sizes (e.g. Cohen's *d*, Pearson's *r*), indicating how they were calculated

Our web collection on statistics for biologists contains articles on many of the points above.

Software and code

Policy information about availability of computer code

Data collection: No software was used.

Data analysis: The BL3.2-CTX interface was characterized by EpiC platform (Raven Biosciences, version 0.9), and ColabFold in combination with classical molecular dynamics simulations using the AMBER 22 simulation software package which contains the pmemd.cuda module.

BLI data was processed and analyzed using Octet® Analysis Studio 12.2.2.26 (ForteBio)

The Protein Thermal Shift Software (version 1.4) from Applied Biosystems was used to calculate the Derivative curve determined Tm.

The cAMP standard curve was measured in triplicates and levels of intracellular cAMP interpolated using GraphPad Prism version 9.5.0. Relative IC50 for BL3.2 was determined using a GraphPad Prism version 9.5.0.

CLC Main Workbench version 23.0.2 was used for sequence analysis and alignment.

All other data analysis was performed on GraphPad Prism v10.

For manuscripts utilizing custom algorithms or software that are central to the research but not yet described in published literature, software must be made available to editors and reviewers. We strongly encourage code deposition in a community repository (e.g. GitHub). See the Nature Portfolio guidelines for submitting code & software for further information.

Data

Policy information about availability of data

All manuscripts must include a data availability statement. This statement should provide the following information, where applicable:

- Accession codes, unique identifiers, or web links for publicly available datasets
- A description of any restrictions on data availability
- For clinical datasets or third party data, please ensure that the statement adheres to our policy

The BL3.1 and BL3.2 protein sequence data used in this study are available in the Mendeley database (https://data.mendeley.com/datasets/thvh9j7hbk/1). The mass spectrometry proteomics data generated in this study have been deposited to the ProteomeXchange Consortium via the PRIDE partner repository under accession code PXD057713. Source data are provided with this paper.

Research involving human participants, their data, or biological material

Policy information about studies with human participants or human data. See also policy information about sex, gender (identity/presentation), and sexual orientation and race, ethnicity and racism.

Reporting on sex and gender	n/a
Reporting on race, ethnicity, or other socially relevant groupings	n/a
Population characteristics	n/a
Recruitment	n/a
Ethics oversight	n/a

Note that full information on the approval of the study protocol must also be provided in the manuscript.

Field-specific reporting

Please select the one below that is the best fit for your research. If you are not sure, read the appropriate sections before making your selection.

☒ Life sciences ☐ Behavioural & social sciences ☐ Ecological, evolutionary & environmental sciences

For a reference copy of the document with all sections, see nature.com/documents/nr-reporting-summary-flat.pdf

Life sciences study design

All studies must disclose on these points even when the disclosure is negative.

Sample size	The sample size for the in vivo assays varied from a maximum of 15 animals (infant mice) per group to a minimum of 5 animals per group to ensure statistical significance. The sample size is given for each experiment.
Data exclusions	No excluded data
Replication	The in vivo experiments were not replicated. As we did not know how big the differences between the groups would be, we started out with relatively small cohorts (5-6 animals each). However, the initial experiments showed such a strong phenotype that we were able to calculate statistical significance with the relatively small numbers and did not need further animals. In line with our ethics guidelines, we did not add more animals to the groups or replicated the experiment.
Randomization	Infant mice litters were mixed prior to the start of the experiment
Blinding	Not relevant in toxin neutralization studies as the outcome (acute diarrhea) is clearly evident, especially when using food colouring.

Reporting for specific materials, systems and methods

We require information from authors about some types of materials, experimental systems and methods used in many studies. Here, indicate whether each material, system or method listed is relevant to your study. If you are not sure if a list item applies to your research, read the appropriate section before selecting a response.

Materials & experimental systems

n/a	Involved in the study
☐	☒ Antibodies
☐	☒ Eukaryotic cell lines
☒	☐ Palaeontology and archaeology
☐	☒ Animals and other organisms
☒	☐ Clinical data
☒	☐ Dual use research of concern
☒	☐ Plants

Methods

n/a	Involved in the study
☒	☐ ChIP-seq
☒	☐ Flow cytometry
☒	☐ MRI-based neuroimaging

Antibodies

Antibodies used

The single-domain antibody library used in this study was prepared for Bactolife A/S from two Alpacas immunized with cholera toxin B-subunit at the VIB Nanobody Core (Brussels, Belgium). The anti-CTX VHH used as a benchmark in this study was originally published by Goldman et al. (2006) and full referencing is provided in the manuscript.

For Figure 1a, 360 nM of VHH was used.
For Figure 1b, 36-3600 nM of BL3.1/BL3.2/anti-CTX VHH benchmark were used.
For Figure 2a, BL3.2 was mixed with either SGF, SIF, or PBS (untreated control) to a final concentration of 100 µg/ml.
For Figure 2b, 10 µg of BL3.2 was used.
For Figure 3, various dilutions (0.240–31.25 nM) of BL3.2 and the anti-CTX benchmark were used.
For Figure 5, 250 nM of BL3.1 was used.
For Figure 6 and 7, 9 mg/ml of BL3.2 was used.

No commercial antibodies were used.

Validation

The pSANG10-3F vector of E. coli clones expressing selected VHHs (CTXB–GM1 blocking capacity >25%) was sequenced (Eurofins Genomic, Germany) using primer pBDS100-1 (GTATGTTGTGTGGAATTGTGAGC). The anti-CTX VHH used as a benchmark in this study was originally published by Goldman et al. (2006) and full referencing is provided in the manuscript.

Eukaryotic cell lines

Policy information about cell lines and Sex and Gender in Research

Cell line source(s)

The HCA-7 Cell Line (Product no. C0009003) from AddexBio, supplied by Gentaur Genprice, was used.

Authentication

Not authenticated

Mycoplasma contamination

Tested. No contamination.

Commonly misidentified lines (See ICLAC register)

No commonly misidentified lines.

Animals and other research organisms

Policy information about studies involving animals; ARRIVE guidelines recommended for reporting animal research, and Sex and Gender in Research

Laboratory animals

Immunization of two alpacas and blood collection was performed by the VIB Nanobody Service Facility (Brussels, Belgium). Animal experiments were conducted in 5-day old CD-1 infant mice (5-day old). Mice were supplied by Charles River Laboratories. Litters mixed before experiment start.

Wild animals

No wild animals involved in this study

Reporting on sex

This information was not collected as it was not considered relevant for this study.

Field-collected samples

n/a

Ethics oversight

Immunizations and handling of the animals were performed according to directive 2010/63/EU of the European parliament for the protection of animals used for scientific purposes and approved by the Ethical Committee for Animal Experiments of the Lamasté (permit No. 2020.1_NSF).

The mice experiments were performed according to a protocol (2016N000416) reviewed and approved by the Brigham and Women's Hospital Institutional Animal Care and Use Committee and in compliance with the Guide for the Care and Use of Laboratory Animals.

The alpaca immunization...

Note that full information on the approval of the study protocol must also be provided in the manuscript.

Plants

Seed stocks	n/a
Novel plant genotypes	n/a
Authentication	n/a
